# Autophagy Delays Apoptotic Cell Death Induced by *Siniperca chuatsi* Rhabdovirus in Epithelioma Papulosum Cyprinid Cells

**DOI:** 10.3390/v13081554

**Published:** 2021-08-06

**Authors:** Guang-Zhou Zhou, Jun Li, Yan-He Sun, Qin Zhang, Lu Zhang, Chao Pei

**Affiliations:** 1 College of Bioengineering, Henan University of Technology, Zhengzhou 450001, China; lj20209537@163.com (J.L.); sunyanhegryx@163.com (Y.-H.S.); zhanglu@haut.edu.cn (L.Z.); 2 Henan Academy of Fishery Sciences, Zhengzhou 450044, China; qinz07@163.com; 3 College of Fisheries, Henan Normal University, Xinxiang 453007, China; peichao@htu.edu.cn

**Keywords:** fish rhabdovirus, autophagy, apoptosis, crosstalk, virus replication

## Abstract

Autophagy and apoptosis are two key cell fate determination pathways, which play vital roles in the interaction between viruses and host cells. Previous research had confirmed that one strain of fish rhabdoviruses, *Siniperca chuatsi* rhabdovirus (SCRV), could induce apoptosis and autophagy after infection. In the current study, we continued to analyze the interaction of autophagy and apoptosis in SCRV-infected EPC cell lines after treatment with different autophagy or apoptosis inhibitors. We found that SCRV infection could activate the mitochondrial apoptotic pathway by the detection of the activities of the caspase-3 and caspase-9 and by flow cytometry analysis in JC-1-stained cells, respectively. Furthermore, no significant autophagy-related factors were disturbed in SCRV-infected cell after apoptosis inhibitor Z-VAD-FMK treatment, while autophagy inducer rapamycin could obviously delay the occurrence of CPE and cell death. Meanwhile, rapamycin was able to reduce the proportion of apoptotic cells. Besides that, rapamycin could disturb the expression of p62 and LC3B-II, and the transcription level of SCRV nucleoprotein mRNA. The progeny virus titers did not show a big difference between the rapamycin treatment or without it. Collectively, our data preliminarily confirmed that SCRV-activated autophagy could delay apoptosis in EPC cells and may not affect virus production. Further study may need to focus on the crosstalk regulation and its roles on the SCRV infection.

## 1. Introduction

Apoptosis and autophagy are two conservative cellular fate-determining manners of eukaryotic cells [1]. Apoptosis is characterized by a number of typical morphological changes in the structure of the cell, together with large amounts of enzyme-dependent biochemical processes. As an active type I programmed cell death pathway adopted by cells after encountering internal and external environmental factors, apoptosis is often regulated by caspases family factors [2], while autophagy, a highly conservative “self-eating” process, can not only remove damaged organelles in cells, degrade macromolecular proteins, maintain cell homeostasis and material recycling, but also drive cell death under physiologically relevant circumstances [3].

In fact, autophagy has been shown to engage in complex interplay with apoptosis in recent years. Autophagy can not only block the induction of apoptosis by inhibiting the activation of apoptosis-associated caspases, but also help to induce apoptosis [4]. Alternatively, the activation of apoptosis-related proteins can also inhibit autophagy by degrading autophagy-related proteins, such as Beclin-1 [5]. For example, Maejima et al. [6] found that a proapoptotic kinase, Mst1, could phosphorylate the BH3 domain of Beclin-1, which forms a protein complex with Bcl-2 and/or Bcl-XL. This phosphorylation inhibited the PI3K activity of the Atg14L-Beclin-1-Vps34 complex and suppressed autophagy. In addition, Beclin-1 could be cleaved by caspase-3 and the C-terminal fragment (Beclin-1-C) could sensitize the cells to apoptosis [7,8]. These studies all highlight the intricate interplay between autophagy and apoptosis.

The rhabdoviridae is a diverse family of negative-sense single-stranded RNA viruses which can infect mammals, birds, reptiles, insects and plants. Presently, the biological features and genetic diversity of some fish rhabdovirus isolates were identified in the past few years [9]. As one kind of important viral pathogens, fish rhabdoviruses could infect several kinds of fish and activate the typical programmed cellular death pathway of fish cells [10]. Present studies indicated that fish rhabdoviruses could induce apoptosis and autophagy by activating several signaling pathways in different cell types [11,12,13]. Du et al. [14] found that one strain of fish rhabdovirus, *Scophthalmus maximus* rhabdovirus (SMRV), could activate caspase-mediated apoptosis in carp leucocyte cells, while another fish rhabdovirus, spring viraemia of carp virus (SVCV), can utilize the autophagy pathway activated by its glycoprotein to facilitate own genomic RNA replication and enhance its titers in the supernatants of *epithelioma papulosum cyprini* (EPC) cells [13]. These studies suggested that the roles of apoptosis and autophagy were very complicated in fish rhabdovirus-infected cells.

*Siniperca chuatsi* rhabdovirus (SCRV) is one of the piscine rhabdoviruses belonging to the Vesiculovirus, which was isolated and identified from cultured diseased turbot *Scophthalmus maximus Linnaeus* [15]. Therefore, it is important to clarify the infection and pathogenic mechanism of SCRV. SCRV could activate apoptosis of EPC cells and induce caspase-mediated cleavage of its nucleoprotein [16]. However, this cleavage did not have sufficient impact on the production of SCRV progeny virus. Additionally, our recent research supports the autophagy activation in EPC cells after SCRV infection. Moreover, autophagy inhibitor (wortmannin) treatment could lead to an increase in CPE in SCRV-infected EPC cells, which suggests the potential existence of interplay between apoptosis and autophagy after infection.

Therefore, here, we continue to explore the role of apoptosis and autophagy in the context of SCRV infection in EPC cells and clarify the specific apoptotic signaling pathway. Moreover, in the presence of an autophagy inducer (rapamycin), this project aims to confirm the limitation of the cytopathic effect (CPE) progress of SCRV in vitro. It is suggested that SCRV-activated autophagy might play a protective role on the process of infection in EPC cells. Current work will no doubt provide theoretical support for future treatment, development of prevention and control technology of fish rhabdovirus diseases.

## 2. Materials and Methods

### 2.1. Cell Line, Virus and Chemicals

EPC cells were cultured with M199 (Procell, USA) culture medium containing 10% FBS (Everygreen, Hangzhou, China) in a 25 cm^2^ culture flask. SCRV virus was stored in a −80 °C refrigerator in our laboratory. The virus titer (TCID_50_) was measured in a 96-well plate using the traditional Reed–Muench method.

3-Methyladenine (3-MA), rapamycin (RAPA) and Z-Val-Ala-Asp(OMe)-FMK (Z-VAD (OME)-FMK) were all obtained from MedChemExpress (MCE, New Jersey, USA). Apoptosis inducer was bought from Biyuntian Co. Ltd. (Shanghai, China). The Annexin V-FITC/PI detection Kit was acquired from Yeasen Biotech Co., Ltd. A TUNEL detection Kit (IV-CY3) was purchased from Boster Biological Technology Co. Ltd. (Wuhan, China). Anti-LC3B, anti-p62, anti-GAPDH and secondary antibodies were purchased from Sigma (Shanghai, China), and anti-caspase-3 antibody was purchased from Proteintech Group, Inc. The reverse transcription kit (Hifair II 1st Strand cDNA Synthesis SuperMix for qPCR) used in this study was bought from Vazyme Biotech Co., Ltd. (Nanjing, China).

### 2.2. CPE Observation

A total of 6 × 10^5^ cells/well was seeded in a 6-well plate and subcultured to a single layer. EPC cells were co-treated with SCRV (300 TCID_50_) and different chemicals (3-MA, rapamycin or Z-VAD-FMK), respectively. At 12 h, 24 h, 36 h and 48 h, the effects of different chemical on the CPE of the infected cells were observed under the inverted microscope and pictures were taken by a photographer (Canon, Tokyo, Japan).

### 2.3. Fluorescent Dye Staining

An amount of 1 × 10^5^ cells/well were inoculated and cultured for 24 h on a 24-well plate, and then infected with SCRV (300 TCID_50_) for another 24 h. After washing with precooling PBS twice, cells were incubated with three different fluorescent dyes including acridine orange (AO, 0.01 mg/mL, 40 s), dansylcadaverine (MDC, 0.05 mM, 10 min) or DAPI (1 μg/mL, 10 min), respectively, which were applied to catch the autophagic and apoptotic signals in the SCRV-infected cells. The stained cells were finally photographed with a fluorescence microscope (Nikon Eclipse TS200-U, Tokyo, Japan) at 380 nm.

For autophagy detection, 5 × 10^4^ cells/well were seeded on a 24-well plate. After coming to 70% confluence, cells were transfected with pEGFP-LC3 recombinant DNA with Lipofectamine^2000^ according to the manufacturer’s protocol (Invitrogen, Carlsbad, CA, USA). One day later, cells were infected with SCRV (300 TCID_50_) for another 24 h. Hoechst 33342 was next used to stain the nuclei. Pictures were taken again by the fluorescence microscope.

### 2.4. Electron Microscopy

The SCRV-infected EPC cells were collected and performed for electron microscope detection as described previously [15]. The grids containing ultrathin sections were examined with a JEM-1230 electron microscope and micrographs were taken with a charge-coupled device (CCD) camera.

### 2.5. TUNEL Detection

Pre-treated coverslips were placed in a 24-well plate and EPC cells were then inoculated at a density of 1 × 10^5^ per well. Subsequently, the cells were treated with SCRV dilution and chemicals (3-MA, Z-VAD-FMK and rapamycin) for 24 h at 25 °C, respectively. Next, the cells were fixed with 4% paraformaldehyde at room temperature for 30 min, and were labeled for 2 h with labeling buffer containing TDT and DIG-dUTP at 37 °C. After sealing for 30 min at 25 °C, the samples were accordingly incubated with anti-digoxin antibody and SABC antibody (1:100) at 37 °C. DAPI was used to dye the nuclei. Samples were observed and photographed under the confocal microscope (Olympus FV3000, Tokyo, Japan).

### 2.6. Flow Cytometry Analysis

Annexin V-FITC/PI double staining was employed to detect the proportion of apoptotic cells in the infected samples. In brief, after inoculating and treating the cells with inhibitors or SCRV dilution for 24 h, the cells were collected carefully and stained according to the kit protocol, respectively. The percentage of apoptotic cells were counted by flow cytometry (FACS Calibur, BectonDickinson, Franklin Lakes, NJ, USA).

### 2.7. Caspase Activity Assay

EPC cells were co-treated with SCRV dilution and different chemicals (3-MA, Z-VAD-FMK and rapamycin) for 24 h and 48 h, respectively. The activities of caspase-3, -8 and -9 were analyzed according to the protocol of the enzyme activity kit (Beyotime, Shanghai, China). Briefly, the cell supernatants were collected, and their protein concentrations were measured. Then, the reaction system was prepared according to the instructions and incubated at 37 °C for 2 h. The samples were analyzed using an enzyme labeling instrument at 405 nm. The enzyme activities were calculated by comparing the standard curve and protein concentration.

### 2.8. Mitochondrial Membrane Potential (MMP) Analysis

The values of MMP were assayed by 5,5’,6,6’-Tetrachloro-1,1’,3,3’-tetraethylbenzimidazolylcarbocyanine iodide (JC-1) staining according to usual procedures. Briefly, the treated EPC cells were collected, and washed with PBS, followed by incubating with JC-1 (10 mM) for 30 min at 37 °C in the dark. Then, cells were analyzed with the flow cytometry.

### 2.9. Measurement of Reactive Oxygen Species (ROS)

Changes in intracellular ROS level of SCRV-infected EPC cells were determined using the fluorescent marker 2′,7′-dichlorodihydrofluoresceindiacetate (DCFH-DA) staining. Briefly, pre-treated EPC cells were trypsinized, washed and incubated with DCFH-DA (10 mM) for 30 min in the dark. Then, the intensity of fluorescence was measured by flow cytometry and analyzed with CellQuest analysis software.

### 2.10. Western Blotting

After treatment with SCRV dilutions and several chemicals (3-MA, Z-VAD-FMK and rapamycin) for different hours, respectively, the treated EPC cells were collected, and the protein concentrations were determined by the Coomassie brilliant blue method. The proteins were subjected to SDS-PAGE and then transferred to the PVDF membrane for Western blotting. Briefly, after blocking with TBS containing 5% non-fat milk, PVDF membranes were incubated with primary antibodies and the corresponding HRP-conjugated secondary antibodies successively. The target proteins were visualized by typical enhanced chemiluminescence (ECL) kits. A computer-based Image Analysis program (ImageJ version1.53j, https://imagej.nih.gov/ij/) was used to quantify the intensity of each band.

### 2.11. qRT-PCR

The total RNA of treated EPC cells and control group cells was extracted according to the manufacturer’s protocols (Tsingke, Beijing, China). After reverse transcription of cellular RNA into cDNA with standard kit procedures, qPCR reactions were performed with the SCRV N gene primers (forward: ATCCATCAGATCACAGAACGC, reverse: TCCCAGCCATTCTCCTCAGTCC) using the following thermocycling condition: initial denaturation at 95 °C for 1 min followed by 40 cycles of amplification (10 sec at 95 °C, 10 sec at 56 °C and 10 sec at 72 °C). The absorption values of the SYBR Green I in each tube were detected at the end of each cycle. The melting curve analysis of PCR products from 60–95 °C were also performed after amplification. Eighteen s was used as the internal control, whose amplification primers were: forward 5′-CATTCGTATTGTGCCGCTAGA-3′ and reverse 5′-CAAATGCTTTCGCTTTGGTC-3′. The fold change (FC = 2^−ΔΔCt^) in N mRNA expression level was normalized to that of the 18 s transcripts measured with the same cDNAs. The relative N mRNA value in each group was calculated using the 2^−ΔΔCt^ method. Every sample was detected ≥ 3 times.

### 2.12. Statistical Analysis

All statistical analyses were performed with at least three independent experiments. All data were represented as the mean ± standard error (S.E.) and analyzed by IBM SPSS Statistics 22. Significances were shown as *p* < 0.05 (*), *p* < 0.01 (**) and *p* < 0.001 (***).

## 3. Results

### 3.1. SCRV Infection Can Activate Autophagy of EPC Cells

**Figure 1:**
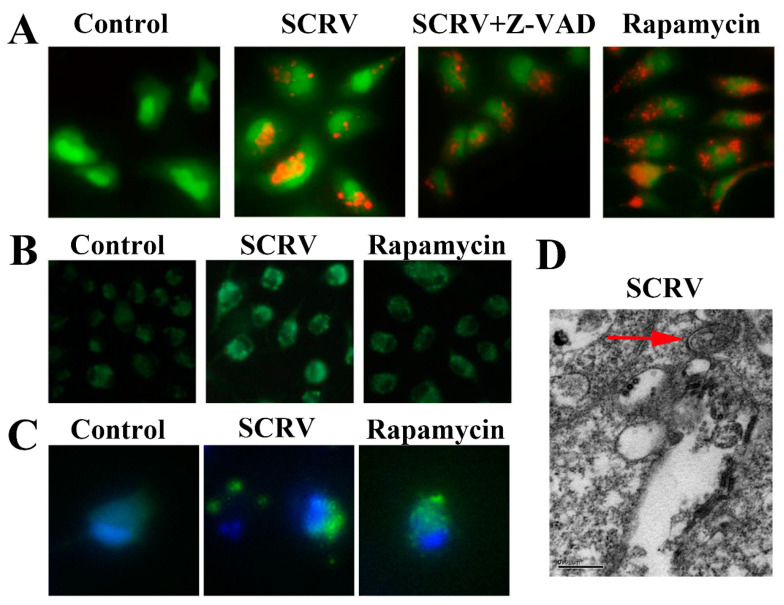
Autophagy induction analysis in SCRV-infected EPC cells. (**A**,**B**) Stand up the acidic vesicles and autophagosome structures of infected cells by AO and MDC staining, respectively. Apoptosis inhibitor Z-VAD-FMK (abbreviated Z-VAD) and autophagy inducer rapamycin were also explored to incubate SCRV-infected cells, respectively. (Original magnification, ×200). (**C**) Punctuate aggregations of LC3 were detected in EPC cells after transfection with recombinant pEGFP-LC3 vector. Hoechst 33342 was used to stain the nuclei. (**D**) Electron microscope observation of autophagic double-membrane structure (denoted by the red arrow) in SCRV-infected cells. (Original magnification, ×80k).

Firstly, we infected EPC cells with SCRV (300 TCID_50_) for 24 h, and then used AO or MDC to stain the infected cells, respectively. The fluorescence microscope could detect the obvious bright red acidic vesicles in the cytoplasm of the treated cells (Figure 1A), while no apparent signals were found in the blank control group. Similarly, as an eosinophilic fluorescent pigment, MDC was used to expose large numbers of dark spot-like aggregation signals in the SCRV-infected group (Figure 1B). We also found the punctuate aggregation of LC3 protein in the cytoplasm of pEGFP-LC3 recombinant vector-transfected cells (Figure 1C). Besides that, the electron microscope was applied to scan the SCRV-infected cells and the typical autophagy double-layer membrane structures were detected in the cytoplasm (Figure 1D). All above results confirmed the activation of autophagy in SCRV-infected EPC cells.

### 3.2. SCRV Infection Can Induce Mitochondrial Apoptosis of EPC Cells

**Figure 2:**
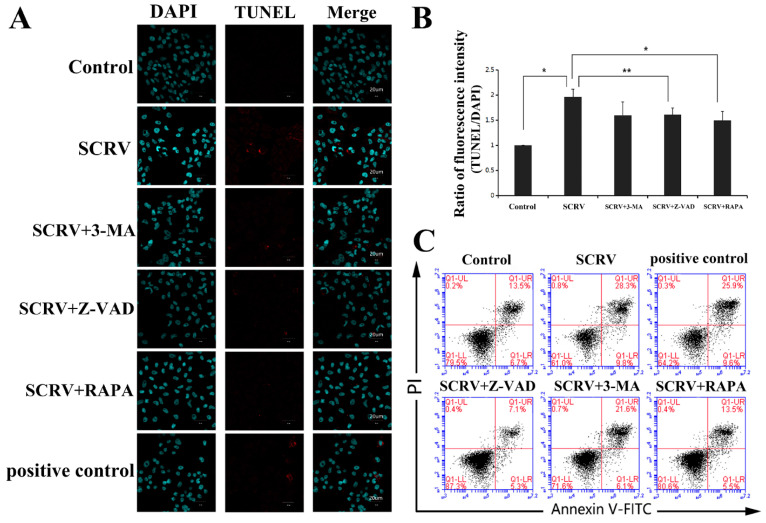
Detection of the effects of different chemical treatments on apoptosis in SCRV-infected EPC cells. (**A**) Immunofluorescence analysis of SCRV-infected cells after DAPI and TUNEL staining at 24 h. The infected cells were co-treated with Z-VAD-FMK (100 μM), 3-MA (5 mM) and rapamycin (1 μM, abbreviated RAPA), respectively. The nuclei were stained with DAPI (blue). The nuclei of apoptotic cells are stained with TUNEL (red). (**B**) Statistical analysis of fluorescence intensity of TUNEL/DAPI. Rapamycin could reduce the value of intensity statistically. *p* < 0.05 (*), *p* < 0.01 (**). (**C**) Flow cytometry analysis of SCRV-infected cells after AV-PI double staining at 24 h. Rapamycin could also reduce the percentage of apoptotic cells.

Simultaneously, we used fluorescent dye (DAPI) to stain the virus-infected cells. The characteristic nuclear condense or fragmentation of the nuclei was detected after SCRV infection (left column in Figure 2A). Moreover, TUNEL analysis demonstrated that the apoptotic cells contracted, and their nuclei appeared navy and the perinuclei red in the SCRV-treated group, whereas the positive signals were not detected in control cells (middle column in Figure 2A). The ratio of fluorescence intensity of TUNEL/DAPI in SCRV infection group was statistically higher than that in control group (Figure 2B). After Annexin V-PI double staining, flow cytometry was used to count the proportion of apoptotic cells in SCRV-infected EPC cells. The percentage of apoptotic cells (red fluorescence intensity/blue fluorescence intensity) was calculated from the whole field of the pictures. The result showed that the number of apoptotic cells in the infection group increased significantly, coming to 38.1% (total percentage of early and late apoptotic cells) at 24 h.p.i (Figure 2C). Alternatively, SCRV-activated apoptosis could be significantly inhibited by the apoptosis inhibitor Z-VAD-FMK. In the SCRV plus Z-VAD-FMK treatment group, the proportion of total apoptotic cells was only 12.4% (Figure 2C). These results confirmed that SCRV induced caspase-dependent apoptosis in EPC cells.

**Figure 3:**
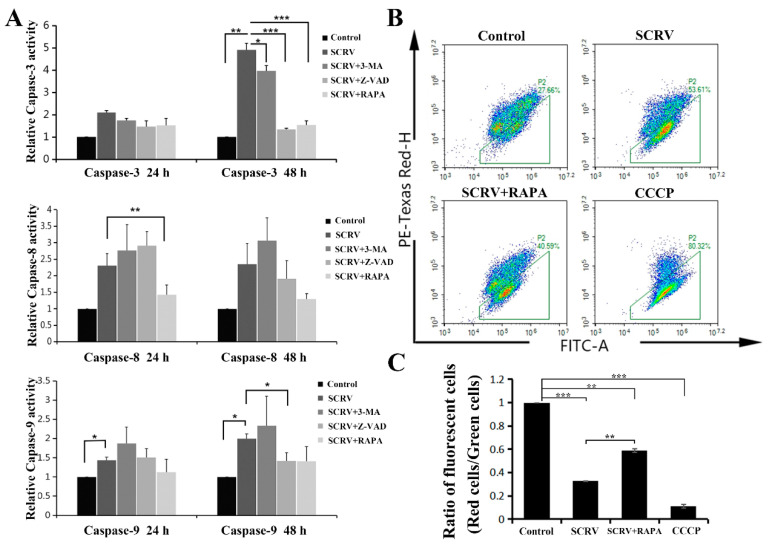
Effects of autophagy or apoptosis inhibitors on the SCRV-infected EPC cells. (**A**) Detection of caspase-3, -8 and -9 activities in SCRV-infected cells at 24 h and 48 h, respectively. The infected cells were co-treated with Z-VAD (100 μM), 3-MA (5 mM) and RAPA (1 μM), respectively. (**B**) Flow cytometry analysis of SCRV-infected cells after rapamycin co-treatment at 48 h. (**C**) Statistical analysis of ratios of fluorescent cells (red cells/green cells in Figure 3B), which suggested that rapamycin could reduce the proportion of apoptotic cells, comparing with SCRV-only infection group. *p* < 0.05 (*), *p* < 0.01 (**) and *p* < 0.001 (***).

The caspase family is closely related to cell apoptosis. In order to further identify the specific apoptotic pathway induced by SCRV in EPC cells, the activities of several caspases (including caspase-3, -8 and -9) were detected. Current results exhibited a sharp increase in caspase-3 and caspase-9 activity at 24 h and 48 h, respectively, while no significant change in caspase-8 was found (Figure 3A). The activity of caspase-3 increased by about five times comparing with the control group at 48 h.p.i. Meanwhile, Z-VAD-FMK could mitigate this increase in enzyme activities at different time points (Figure 3A). This suggested that SCRV might activate the effector caspase-3 by inducing the activation of caspase-9 in EPC cells, and then initiating the caspase cascade of mitochondrial apoptosis. Moreover, the reduction in mitochondrial membrane potential (MMP) could be seen as a landmark event of the early stage of apoptosis. Hence, we used JC-1 (one fluorescent probe) to stain the treated cells and detected the MMP. JC-1 could produce red light in the matrix with high MMP, and green light when the potential is low. The present results showed that the ratio of green/red intensity in SCRV-infection group was significantly higher than that in control cells, coming to 53.61% (Figure 3B,C). These data further suggested that SCRV might induce the mitochondrial apoptosis pathway of EPC cells.

### 3.3. Autophagy Can Combat the Apoptosis of EPC Cells after SCRV Infection

In Figure 2B, we have used the TUNEL assay to evaluate the EPC cells apoptosis after SCRV infection. Interestingly, in addition to Z-VAD-FMK, another autophagy inducer, rapamycin, could also resist the induction of apoptosis in SCRV-infected EPC cells. In the infected cells, rapamycin could reduce the red apoptotic signals in the TUNEL assay (Figure 2A,B). Besides that, flow cytometry analysis of AV/PI double-stained cells also confirmed the decline of apoptotic cells in SCRV-infected group (Figure 2C), whose proportion came to 19.0% in EPC cells. These results preliminarily proved that induction of autophagy can combat the occurrence of apoptosis and promote the survival of EPC cells.

**Figure 4:**
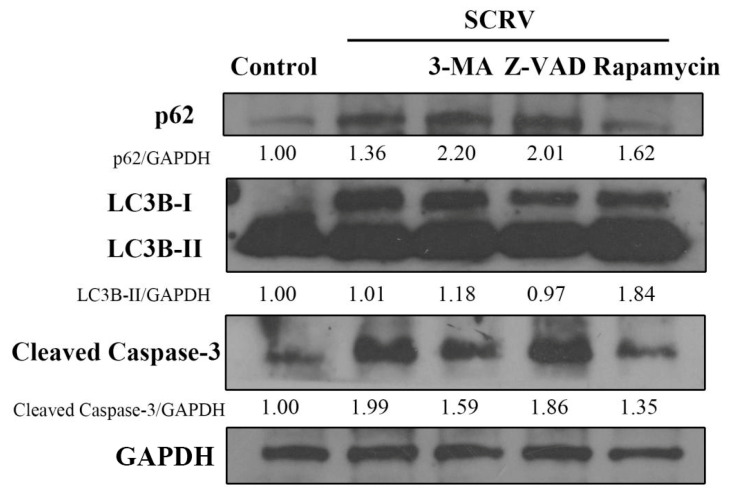
Effects of different chemicals (3-MA, Z-VAD and rapamycin) on the expression of p62, LC3 and caspase-3. Western blotting analysis showed that rapamycin could reduce the expression level of p62 and caspase-3 in SCRV-infected EPC cells at 24 h. GAPDH was used as internal control.

Furthermore, Western blotting analysis was carried out to confirm the crosstalk relationship between autophagy and apoptosis induced by SCRV in EPC cells. The results showed the increase in autophagy marker protein LC3B-II (Figure 4), which further demonstrated the activation of autophagy in SCRV-infected cells. Interestingly, another autophagy marker factor, the expression of p62, also increased at 24 h, which suggested the blockage of autophagic flux. In addition, there is no notable statistical expression difference of LC3B and p62 when pretreated with Z-VAD-FMK in virus-infected cells. However, the autophagy inducer, rapamycin, could obviously inhibit expression of the apoptosis-related protein, caspase-3. This further suggested that autophagy could have an impact on the apoptosis in SCRV-infected EPC cells.

### 3.4. Effects of Apoptosis and Autophagy on the Infection of SCRV in EPC Cells

**Figure 5:**
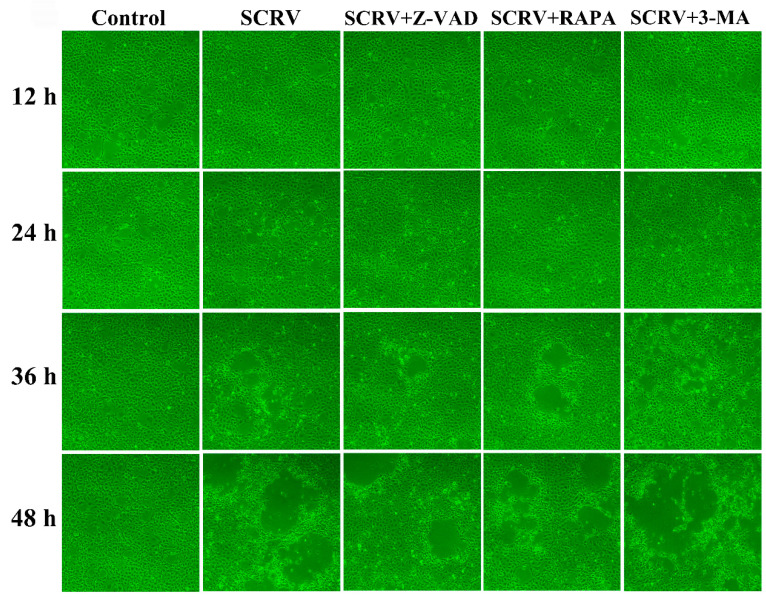
CPE observation of SCRV-infected EPC cells after co-incubation with different chemical (3-MA, Z-VAD and RAPA). Rapamycin could postpone the progress of CPE induced by SCRV at different time points. (Original magnification, ×100).

In order to study the impact of autophagy and apoptosis on SCRV replication and cell proliferation in EPC cells, we observed the occurrence of CPE after Z-VAD-FMK and rapamycin treatment at different time points, respectively. At 24 h, the cell monolayer was better in the SCRV plus Z-VAD-FMK or rapamycin-pretreated group than that in the positive infection group. Moreover, less CPE were captured after microscope detection at 48 h (Figure 5). Subsequently, our results suggested that both Z-VAD-FMK and rapamycin could effectively delay the emergence of CPE and cell death caused by SCRV infection. On the contrary, autophagy inhibitor treatment (3-MA) could help CPE to progress in SCRV infection, and most of the cell monolayer had been broken (Figure 5).

**Figure 6:**
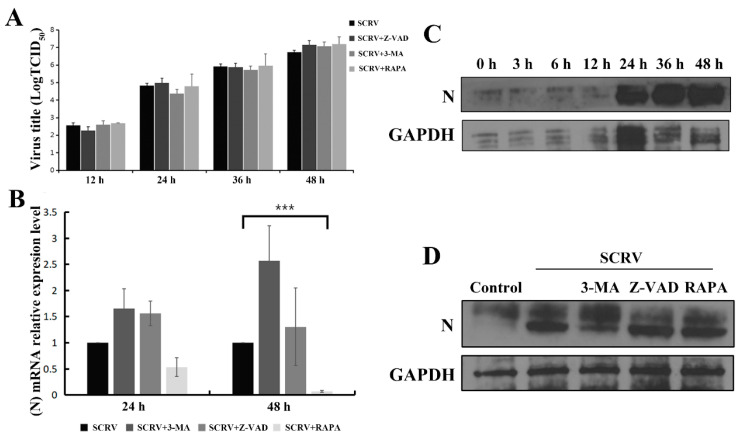
Impacts of autophagy and apoptosis on the progeny virus replication and expression of SCRV N protein in EPC cells. Infected EPC cells were co-treated with Z-VAD (100 μM), 3-MA (5 mM) and RAPA (1 μM), respectively. (**A**) The virus titers in the cell supernatants were detected at 12 h, 24 h, 36 h and 48 h. (**B**) qRT-PCR was used to analyze the SCRV N gene mRNA level and rapamycin treatment could significantly reduce the level of N mRNA in SCRV-infected cells. *p* < 0.001 (***). (**C**) Western blotting analysis was applied to assess the viral N protein expression after SCRV infection at different time points. GAPDH was used as internal control. (**D**) Western blotting analysis of N protein expression was carried out after different chemical treatments in SCRV-infected EPC cells at 48 h.

The above results had shown that apoptosis and autophagy could play an important role in cell death after SCRV infection in EPC cells; here, we continued to identify whether the progeny virus titers were affected or not in the above-described different treatment conditions. Interestingly, perturbing apoptosis or inducing autophagy has little effect on the SCRV progeny virus titers in the cell supernatants at 12 h, 24 h, 36 h and 48 h (Figure 6A), and there is no statistical significance of virus load in several groups. This suggested that replication of the progeny SCRV might not be affected by cell autophagy or apoptosis at current set time points.

### 3.5. Impacts of Apoptosis and Autophagy on the SCRV N Protein

Rhabdovirus nucleoprotein (abbreviated N) is a key player for the replication and assembly of viruses. Herein, we continued to explore the impacts of autophagy or apoptosis on the N mRNA level and its protein expression after different chemical treatment in SCRV-infected EPC cells. Present qRT-PCR analysis indicated that rapamycin could reduce the N gene mRNA level of SCRV at 48 h, revealing significant statistical differences with that of virus-infection group (Figure 6B). Moreover, expression of SCRV N protein exhibited a gradual increase trend in a time-dependent manner from 0 h to 48 h (Figure 6C). Unexpectedly, no apparent change of N expression level was detected after rapamycin treatment (Figure 6D). Future work should focus on the post-transcriptional modification or translation of the N protein of SCRV-infected EPC cells after different chemical treatments.

## 4. Discussion

Outbreaks of fish rhabdovirus diseases have become a big threat to aquaculture and led to great economic losses. So far, many rhabdovirus strains have been isolated and identified from wild or farmed fish bodies around the world in recent decades [17]. Revealing the infection and pathogenic mechanisms of fish rhabdoviruses is vital for the construction of antiviral techniques and development of vaccine or antiviral drugs in the future [18], as one strain of fish rhabdovirus, SCRV, could infect some kinds of fish and activate the typical programmed apoptotic cell death [19]. Recent research confirmed that it could activate autophagy via the PI3K/Akt-mTOR pathway in one fish cell line, Chinese perch brain (CPB) [20]. These works obviously will help to elucidate the pathogenic mechanism of fish rhabdoviruses.

In this study, we further confirmed that SCRV infection could induce autophagy in EPC cell lines by AO or MDC staining and electron microscope observation, respectively. Meanwhile, the apoptotic pathway activated by SCRV in EPC cells was explored by detecting the enzyme activities of different caspases, including caspase-3, caspase-8 and caspase-9. It is well-known that apoptosis pathways mainly included the extrinsic and the intrinsic mitochondrial and the intrinsic ER pathway. The present study suggested that caspase-3 and caspase-9 activities possessed an apparent increase compared with the control group. Additionally, after JC-1 staining, flow cytometry analysis further confirmed the reduction in the mitochondrial membrane potential of the virus-infected cells. The above data suggested that SCRV might induce mitochondrial apoptotic pathway.

In fact, as two key cell fate determination pathways, autophagy and apoptosis have important roles in the interactions between viruses and host cells. Many researchers have focused on the effects of virus-induced autophagy and apoptosis on the viral replication cycle and cellular antiviral immune process and obtained much progression on this subject with different virus strains [2]. Studies have found that host cells could use apoptosis and autophagy to limit the amplification and transmission of invading viruses. In turn, the virus can produce more offspring viruses or facilitate its own release in the process. Recently, more deep research progress on the crosstalk between the autophagy and apoptosis induced by different viruses was acquired, such as viral families including Herpesviridae, Flaviviridae, Orthomyxoviridae, Paramyxoviridae, etc. [2] For instance, Zhou et al. [21] found that autophagy could postpone apoptotic cell death through Bad-Beclin-1 interaction in PRRSV-infected MARC-145 cells, while Chikungunya virus-induced autophagy delays caspase-dependent cell death [22]. Besides that, researchers also found similar results in HSV-1-infected U251 glioma cells, Newcastle disease virus-infected chick embryo fibroblasts cells and Respiratory syncytial virus-infected HEp-2 cells [23,24,25].

Therefore, for one important rhabdovirus pathogen in aquaculture, it is necessary to understand the molecular process and investigate the mechanism mediating the crosstalk between autophagy and apoptosis in SCRV infection. Herein, we applied autophagy and apoptosis inhibitors to treat SCRV-infected EPC cells, and then analyzed the changes of apoptotic and autophagic factors, respectively. We determined that autophagy was activated in EPC cells upon infection with SCRV, while cell apoptosis could be induced at the later stage of viral infection, indicating that there exists a time difference between SCRV-induced autophagy and apoptosis. The present results showed that autophagy could postpone apoptotic cell death in SCRV-infected EPC cells. Research indicated that the autophagy activator (rapamycin) or the apoptosis inhibitor (Z-VAD-FMK) could both delay the SCRV-infected EPC cell death. Moreover, rapamycin reduced the positive apoptotic signals in SCRV-infected EPC cells by TUNEL assay. Flow cytometry analysis detected fewer proportions of apoptotic cells in SCRV-infected cells after rapamycin treatment. Besides that, Western blot analysis indicated that rapamycin could inhibit the expression of caspase-3, the apoptotic marker protein, while in turn, Z-VAD-FMK could not produce statistical influence on the autophagy in SCRV-infected EPC cells, which further suggested that SCRV-activated autophagy occurred before apoptosis in EPC cells.

Furthermore, we explored the impact of autophagy and apoptosis on the replication of the SCRV progeny virus. Although Z-VAD-FMK and rapamycin both delayed the CPE in SCRV-infected EPC cells, there are no statistical differences of SCRV progeny virus titers in cell supernatants between different chemical plus virus treatment groups and normal infection group from 12 h to 48 h. Similar results were also obtained with cell lysates of the above groups (data not shown). In fact, our previous study on the influence of Z-VAD-FMK on SCRV replication had shown that it did not alter the amplification of SCRV, indicating that caspase activation was not required for SCRV replication in EPC cells [16]. The current data was consistent with previous reports. On the other hand, rapamycin postponed the occurrence of CPE and cell death in SCRV infection, but it might not influence the progeny virus replication in infected cells, which then led to the statistically undifferentiated virus titer determination.

Collectively, our current study is the first report on the crosstalk of autophagy and apoptosis induced by fish rhabdovirus, which preliminarily revealed the delay of apoptosis by autophagy in SCRV-infected EPC cells. This research provides a novel insight into SCRV–host interactions and the discovery of new antiviral strategies or drugs against rhabdovirus infection by targeting autophagy.

## Data Availability

Raw data are publicly available at https://www.mdpi.com/ethics.
